# *Lactobacillus gasseri* prevents ibrutinib-associated atrial fibrillation through butyrate

**DOI:** 10.1093/europace/euaf018

**Published:** 2025-01-17

**Authors:** Ling Shi, Yu Duan, Ning Fang, Ning Zhang, Sen Yan, Kunna Wang, Te Hou, Zhiqi Wang, Xiaohui Jiang, Qianhui Gao, Song Zhang, Yue Li, Yun Zhang, Yongtai Gong

**Affiliations:** Department of Cardiology, The First Affiliated Hospital, Harbin Medical University, Youzheng Street 23#, Nangang District, Harbin 150001, China; Department of Cardiology, The First Affiliated Hospital, Harbin Medical University, Youzheng Street 23#, Nangang District, Harbin 150001, China; Department of Cardiology, The First Affiliated Hospital, Harbin Medical University, Youzheng Street 23#, Nangang District, Harbin 150001, China; Department of Cardiology, The First Affiliated Hospital, Harbin Medical University, Youzheng Street 23#, Nangang District, Harbin 150001, China; Department of Cardiology, The First Affiliated Hospital, Harbin Medical University, Youzheng Street 23#, Nangang District, Harbin 150001, China; Department of Cardiology, The First Affiliated Hospital, Harbin Medical University, Youzheng Street 23#, Nangang District, Harbin 150001, China; Department of Cardiology, The First Affiliated Hospital, Harbin Medical University, Youzheng Street 23#, Nangang District, Harbin 150001, China; Department of Cardiology, The First Affiliated Hospital, Harbin Medical University, Youzheng Street 23#, Nangang District, Harbin 150001, China; Department of Cardiology, The First Affiliated Hospital, Harbin Medical University, Youzheng Street 23#, Nangang District, Harbin 150001, China; Department of Cardiology, The First Affiliated Hospital, Harbin Medical University, Youzheng Street 23#, Nangang District, Harbin 150001, China; Department of Cardiology, The First Affiliated Hospital, Harbin Medical University, Youzheng Street 23#, Nangang District, Harbin 150001, China; Department of Cardiology, The First Affiliated Hospital, Harbin Medical University, Youzheng Street 23#, Nangang District, Harbin 150001, China; Department of Cardiology, The First Affiliated Hospital, Harbin Medical University, Youzheng Street 23#, Nangang District, Harbin 150001, China; Department of Cardiology, The First Affiliated Hospital, Harbin Medical University, Youzheng Street 23#, Nangang District, Harbin 150001, China; Key Laboratory of Cardiac Diseases and Heart Failure, Harbin Medical University, Harbin 150001, China; Heilongjiang Key Laboratory for Metabolic Disorder and Cancer Related Cardiovascular Diseases, Harbin Medical University, Harbin 150081, China; Heilongjiang Academy of Medical Science, Institute of Metabolic Disease, Harbin, China

**Keywords:** Ibrutinib, Atrial fibrillation, *Lactobacillus gasseri*, Butyrate

## Abstract

**Background:**

Ibrutinib, a widely used anti-cancer drug, is known to significantly increase the susceptibility to atrial fibrillation (AF). While it is recognized that drugs can reshape the gut microbiota, influencing both therapeutic effectiveness and adverse events, the role of gut microbiota in ibrutinib-induced AF remains largely unexplored.

**Method:**

Utilizing 16S rRNA gene sequencing, faecal microbiota transplantation, metabonomics, electrophysiological examination, and molecular biology methodologies, we sought to validate the hypothesis that gut microbiota dysbiosis promotes ibrutinib-associated AF and to elucidate the underlying mechanisms.

**Result:**

We found that ibrutinib administration pre-disposes rats to AF. Interestingly, ibrutinib-associated microbial transplantation conferred increased susceptibility to AF in rats. Notably, ibrutinib induced a significantly decrease in the abundance of *Lactobacillus gasseri* (*L. gasseri*), and oral supplementation of *L. gasseri* or its metabolite, butyrate (BA), effectively prevented rats from ibrutinib-induced AF. Mechanistically, BA inhibits the generation of reactive oxygen species, thereby ameliorating atrial structural remodelling. Furthermore, we demonstrated that ibrutinib inhibited the growth of *L. gasseri* by disrupting the intestinal barrier integrity.

**Conclusion:**

Collectively, our findings provide compelling experimental evidence supporting the potential efficacy of targeting gut microbes in preventing ibrutinib-associated AF, opening new avenues for therapeutic interventions.

## Graphical Abstract

**Graphical abstract:**
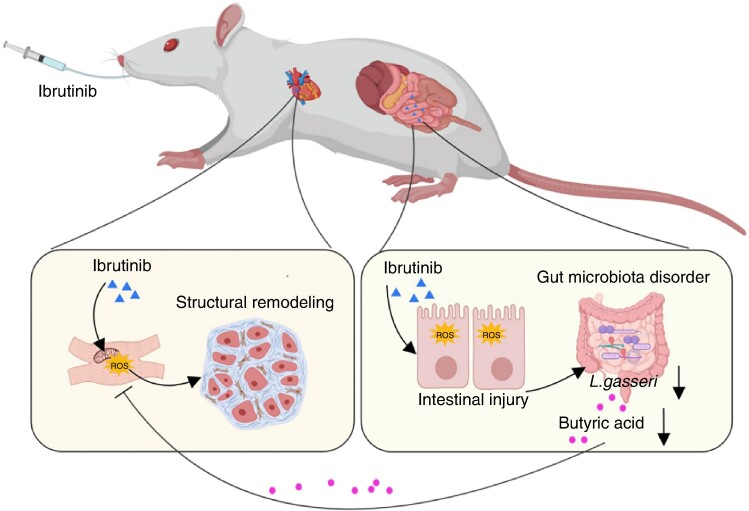

## Introduction

Ibrutinib, a novel and potent Bruton tyrosine kinase inhibitor, has been approved by the U.S. Food and Drug Administration for the management of chronic lymphocytic leukaemia, mantle cell lymphoma, and marginal cell lymphoma.^1^ Despite its efficacy and generally favourable tolerability, the occurrence of atrial fibrillation (AF) is substantially increased in patients undergoing ibrutinib therapy.^2^ AF manifests in as many as 16% of patients receiving ibrutinib over a median follow-up duration of 28 months, thereby potentially posing a limitation to their clinical applications.^3^ Despite the urgent need for effective preventative and therapeutic strategies for ibrutinib-associated AF, the precise mechanisms remain largely unknown.

The human gastrointestinal tract harbours a diverse pool of microbial communities, playing a crucial role in maintaining host health. Once disrupted, the function of intestinal tract and other organ systems will be compromised, pre-disposing the host to a range of diseases, including tumorigenesis and cardiovascular conditions.^4–7^ Mounting evidence reveals that the gut microbiota has been implicated in modulating the efficacy and toxicity of anti-cancer drugs.^8,9^ Anti-cancer drugs may induce gut microbiota dysbiosis, which in turn contributes to their side effects including diarrhoea, intestinal inflammation, colitis, and neurotoxicity.^10–13^ It is also well recognized that microbiota play a key role in the progression of cardiovascular diseases. Our previous study showed that oral administration of *Bacteroides fragilis* prevented rats from aging-related AF.^14^ However, whether gut microbiota is involved in ibrutinib-related AF remains to be determined.

*Lactobacillus* is a common probiotic, which can enhance the efficacy of anti-cancer drugs.^15,16^ Short-chain fatty acid (SCFAs), as important metabolites of *Lactobacillus*, have been reported to improve the chemotherapeutic efficacy of anti-cancer drugs.^17–19^ Furthermore, SCFAs alleviated PD-1/PD-L1 inhibitor-related cardiotoxicity via PPARα-CYP4X1 axis in colonic macrophages.^20^ More importantly, SCFAs exerts cardioprotective property in various cardiovascular diseases, such as atherosclerosis, myocardial infarction, and vascular calcification.^21–23^ Mice with low SCFAs levels exhibited increased vulnerability to AF, which could be alleviated by the supplementation of SCFAs.^24^ So far, the research on the relationship between SCFAs and ibrutinib-related AF remain elusive.

In the present study, we endeavoured to examine the involvement of ibrutinib-induced gut dysbiosis in the aetiology of AF and elucidated the possible underlying mechanism. Our findings revealed that ibrutinib-associated AF can be reversed by *Lactobacillus gasseri* supplementation, primarily attributed to the effective role of *L. gasseri-*derived butyrate (BA) on mitigating ibrutinib-associated oxidative stress in atrial myocytes. Hence, we explored the interaction between ibrutinib-associated AF and gut microbiota dysbiosis, which raises the possibility of selectively targeting microbiota and microbial metabolites as a promising therapeutic strategy for ibrutinib-associated AF.

## Methods

### Experimental animals

The animal experiments in this study were performed in accordance with the Guide for the Care and Use of Laboratory Animals and were approved by the Animal Care and Utilization Committee of Harbin Medical University. For the experimental groups, 8-weeks old male Wistar rats (200–240 g) were purchased from Beijing Vital River Laboratory Animal Technology Co, Ltd (Beijing, China) and housed in SPF conditions with light/dark cycles of 12 h and had access to food and water *ad libitum*. For the experimental groups, rats in the ibrutinib group were orally administered ibrutinib (17 mg/kg/d; Catalent CTS, LLC, USA) for a duration of 4 weeks. The dosage of ibrutinib was determined based on body surface area, as previously described.^25^ Control rats received the vehicle solution in parallel. The vehicle solution was prepared using dimethylsulfoxide (DMSO, Sigma-Aldrich, Natick, MA, USA) as a solvent for dissolving ibrutinib.

### The 16S rRNA sequencing and microbial analysis

Microbial DNA from faecal samples was extracted using the Fecal Genomic DNA Rapid Extraction Kit (GeneBetter, D213) according to the manufacturer’s instructions, then 16S rRNA was amplified at the V3 to V4 region and sequenced with Illumina novaseq6000. The DADA2 in QIIME2 were used to denoise the data after quality control, ASVs are filtered by default with a threshold of 0.005% of all sequences sequenced. Both the Shannon index and Chao1 represent alpha diversity. The principal co-ordinate analysis (PCoA) based on unweighted UniFrac distances reveal beta diversity. Permutational multivariate ANOVA was used to test the differences between groups in PCoA plot.

### Microbiota transplantation

Faeces from control and ibrutinib rats were collected and pooled by group. Each faeces sample (100 mg) was diluted in 2 mL sterile phosphate buffer (PBS, 50 mg stool/1 mL buffer) and homogenized for 5 min until a pasty consistency was reached. After centrifugation for 3 min at 800 *g*, the supernatant was collected. The adult rat was administered with antibiotics (vancomycin 100 mg/kg, neomycin 200 mg/kg, ampicillin 200 mg/kg, and metronidazole 200 mg/kg) for 1 week to depleted the gut microbiota. Faecal microbiota transplantation (FMT) was performed by gavage in accordance with previous study.^4^ Fasting and 1 mL citrate (0.16 mg/mL sodium picosulfate and 51.2 mg/mL magnesium oxide) was administered to rats 24 h before transplantation. Another 2 mL citrate was given to rats 12 h prior to faecal bacteria transplantation. Then, the FMT experiments by oral gavage with 1 mL three times a week for total 6 weeks in rats to re-colonization of transplanted gut microbiota. The rats gavaged with autogenous faecal were referred to the control-FMT group and the rats gavaged with faecal from ibrutinib rats were referred to the ibrutinib-FMT group.

### Electrophysiological study

Atrial fibrillation was performed according to protocols reported previously.^26^ In briefly, rats were anaesthetized with 1% sodium pentobarbital (30 mg/kg) through peritoneal injection, and underwent open-chest surgery. Then, a 1.9-F octapolar electrophysiological catheter was put on the right atrium to deliver programmed stimulation using an automated stimulator interfaced with the data acquisition system (GY6000; HeNan HuaNan Medical Science & Technology Ltd). The AF inducibility was detected through 50 Hz burst pacing, which was applied for 3 s with 12 bursts separated by a 2 s interval. Successful induction of AF was defined as the occurrence of rapid irregular atrial rhythm lasting for 1 s or more. Atrial fibrillation duration was defined as the mean duration of all AF episodes within 60 s in each rat.

### Isolation and culture of primary rat cardiomyocytes and fibroblasts

The primary rat cardiomyocytes (CM) and fibroblasts were isolated from hearts of 1–3 days old neonatal Wistar rats as described previously.^27^ Briefly, after dissection and washes, the ventricle tissue were cut into 1 mm^3^ pieces and digested with 0.25% trypsin. The combined cell suspension was centrifuged and resuspended in DMEM with 10% foetal bovine serum (FBS). After filtration, the isolated cells were centrifugated at 1200 *g* for 5 min. Subsequently, the cardiac cells were resuspended in DMEM with 10% FBS, 1% penicillin (100 IU/mL), and streptomycin (100 mg/mL), which were seeded in six-well plates. Cells were incubated at 37°C in a humidified atmosphere of 5% CO_2_ and 95% air.

### Histological analysis and Masson’s trichrome staining

Sections of fresh samples included ventricular, atrial, and proximal colon from rats were excised and fixed in 4% paraformaldehyde overnight, and then embedded in paraffin. The sections were continuously cut into 5 μm and stained with Haematoxylin and eosin (HE) and Masson’s trichrome staining. The HE staining images were analysed with ImageScope × 64 to quantify the morphology of hearts and proximal colon, and to measure the colonic villi length. The fibrosis was quantified by software (Image-pro plus 6.0, Meida Cybernetics LP). Collagen volume fraction was calculated as collagen area/total area × 100%.

### Tunel staining

Apoptotic cells were detected with an One Step Tunel Apoptosis Assay Kit (Beyotime, C1086) according to the manufacturer’s protocol.^28^ Briefly, After paraffin sections were dewaxed, 20 μg/mL proteinase K was added and incubated at 37°C for 30 min. After PBS washing, the sections were incubated with Tunel reaction mixture for 1 h at 37°C. Next, 50 µL Streptavidin-HRP solution and 100 µL DAB working solution was added dropwise to each sample. Colour development reaction was conducted at room temperature for 10 min. The staining results were observed and captured by immunofluorescence microscope (Zeiss, Jena, Germany).

### MTT assay

MTT Cell Proliferation Assay (Beyotime, C0009S) was used to analyse the cellular viability as described previously.^29^ Cells were cultured on a 96-well plate at 37°C with 5% CO_2_, and then treated with ibrutinib, ibrutinib + BA, or ibrutinib + PA and PBS with five to six biological replicates per group. After 24 h treatment, etrazolium salt (3-(4,5-dimethylthiazol-2-yl)-2,5-diphenyltetrazolium bromide, MTT) was added to the medium and incubated for 4 h at 37°C. Subsequently, the medium was discarded, and 100 μL of DMSO was added to the wells for 10 min at room temperature. Finally, absorbance was measured at wavelength of 570 nm using a microplate reader (Thermo, MA, USA) within 1 h. Statistical analysis is performed using GraphPad Prism 9.0 software. Variables with three groups were analysed by one-way ANOVA followed by Tukey tests. *P* < 0.05 indicated statistical significance between two groups.

### Measurement of cellular ROS in cultured cardiomyocytes and atrial tissues

ROS production was measured using a dihydroethidium (DHE) assay as described previously.^30^ Superoxide generation in cultured CM and frozen section of atrial, liver and lung tissues were analysed using an ROS assay kit (Beyotime, S0033S). Briefly, primary rat CM were seeded in new 12-well plates and pre-treated with vehicle solution, ibrutinib, ibrutinib + BA, or ibrutinib + PA. CM were washed thrice with PBS and then incubated with 10% goat serum for 30 min at room temperature, and then were loaded with DHE at a concentration of 10 μmol/L for 30 min at 37°C. The aforementioned procedures were also conducted on frozen sections of atrial, liver, and lung tissues from rats. Following the incubation of dyes, we executed wash steps exceeding thrice to thoroughly eliminate any excess dye that did not penetrate the cells. After washing with PBS thrice, the fluorescence intensity of ROS was detected with immunofluorescence microplate (Zeiss, Jena, Germany) and fluorescence intensity was analysed with ImageJ. The DHE fluorescence was calculated in each of five randomly selected fields.

### The cultivation and supplementation of *Lactobacillus gasseri*

*Lactobacillus gasseri* was purchased from BNCC (BNCC339385), which was cultured in MRS liquid medium for 24 h at 37°Cin a CO_2_ incubator (Thermo scientific, USA) containing 5% O_2_, 10% CO_2_, and 85% N_2_. The culture was diluted to a final concentration of 1 × 10^9^ CFU/1 mL with anaerobic PBS, which contains 10% glycerol.

### Co-culture of *Lactobacillus gasseri* and Caco-2 cells

Caco-2 cells (Procell Life Science & Technology Co, Ltd) were grown in Caco-2 cell culture media (Minimal Essential Media (Pricella, CM-0050), 20% foetal bovine serum, and 1% P/S) at 37°C and 5% CO_2_. Cells were passaged every 3–4 days in T25 flasks according to protocols. The concentration of *L. gasseri* was determined by cell counting chamber. Cells were co-cultured with *L. gasseri* at multiplicity of infection of 100:1 for 6 h at 37°C in a CO_2_ incubator (Thermo scientific, USA) with 5% O_2_, 10% CO_2_, and 85% N_2_.^31,32^ Following the co-cultivation of *L. gasseri* with Caco-2 cells, the supernatant was aspirated and fully mixed. One microlitre of the supernatant was pipetted onto an ultraviolet-visible spectrophotometer (NANODROP ONE, USA) cuvette for analysis. The OD600 value of the supernatant was determined by UV spectrophotometer.

### Transwell assay

Before the experiment, the upper part of the transwell chamber was pre-coated with mixture (0.5 mL per hole) until the liquid solidified at the normal temperature. The primary cultured cardiac fibroblasts (CFs) were seeded at a density of 2 × 10^4^ cells/mL on the polycarbonate membrane of the transwell in 12-well plates, the CM treated with vehicle solution (DMSO) and ibrutinib 48 h seeded into the lower part of a transwell chamber (pore size of 0.4 µm; Corning Incorporated, Corning, NY, USA), and 0.5 and 1.5 mL DMEM-F12 medium were added to the upper and lower chamber, respectively. The cells were cultured at humidified atmosphere of 95% air with 5% CO_2_ at 37°C, and the medium was changed every other day. After cells cultured in transwells formed a cell layer, the CFs were harvested and the CFs viability and protein expression were detected.

### Quantitative reverse transcription-polymerase chain reaction

The abundance of *L. gasseri* in faecal samples was quantified by quantitative reverse transcription-polymerase chain reaction. Briefly, DNA from the faecal sample is extracted according to the instructions given by the Fecal Genomic DNA Rapid Extraction Kit (GeneBetter, D213). The RNA levels were determined using SYBR Green I incorporation method on ABI 7500 fast Real Time PCR system and the appropriate primer sets (Supplementary material online, *Table S1*).

### Western blot

The total protein (40–60 µg) extracted from atrial tissues or cells, and then were fractionated by SDS–PAGE and transferred onto PVDF membranes. The membranes were blocked with 5% non-fat milk for 1 h at room temperature, and then incubated with anti-Bcl2 (1:500 dilution, abcam, cat#ab196495) and anti-Bax (1:1000 dilution, proteintech, cat#60267-1-Ig), anti-GAPDH (1:1000 dilution, proteintech, cat#60004-1-lg) at 4°C overnight. Then, the membranes were washed with PBS-T (1 L PBS with 1 mL of Tween 20 added) and incubated with the secondary antibody for 1 h. Finally, the membranes were exposed to ECL buffer and chemiluminescent signals were developed with ECL kit and detected by ChemiDoc XRS gel documentation system.

### Permeability of fluorescein isothiocyanate-dextran

Following 8 h of fasting, rats were gavaged with fluorescein isothiocyanate (FITC)-dextran (40 mg/100 g, Sigma-Aldrich) as described previously.^33,34^ Four hours later, blood samples were collected and centrifuged at 1500 *g* at 4°C for 15 min. FITC fluorescence was quantified in a fluorescence microplate reader. The values of plasma FITC-dextran were calculated based on the standard curve of FITC-dextran.

### Statistical analysis

Statistical analysis was performed using GraphPad Prism 9.0 software. Shapiro–Wilk was used for normality test. Continuous variables are expressed as the mean ± standard error of mean or median and inter-quartile range. Categorical variables are represented as numbers and percentages. The comparison between two groups was assessed by Student’s non-paired *t*-test or Wilcoxon (Mann–Whitney *U*) test. Variables with more than two groups were analysed by one-way ANOVA followed by Tukey tests or Kruskal–Wallis followed by a *post hoc* test with Bonferroni adjustment. *P* < 0.05 indicated statistical significance between two groups.

## Results

### Ibrutinib-induced AF is dependent on the gut microbiota

**Figure 1:**
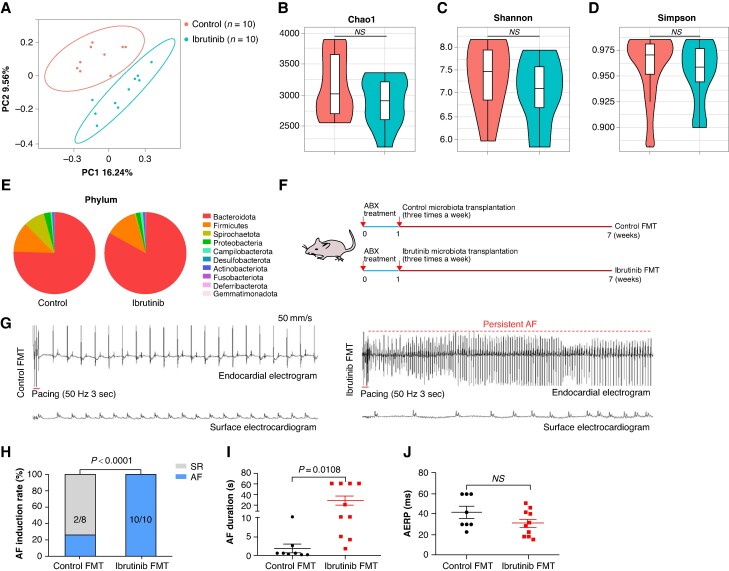
Ibrutinib induces dysbiosis of gut microbiota in rats and promotes atrial fibrillation (AF). (*A*) Principal coordinate analysis (PCoA) of unweighted UniFrac analysis of operational taxonomic units. Each dot represents a single sample of faeces (*n* = 10). (*B–D*) Chao1, Shannon and Simpson indices were analysed in control and ibrutinib. Chao1 indices reflect community richness, and the Shannon and Simpson indices represent community diversity. (*E*) The relative abundance of the microbiome community at the phylum level in control and ibrutinib group. (*F*) Schematic diagram of the experimental design of faecal microbiota transplantation (FMT) in rats. (*G*) Endocardial and surface electrograms recordings in response to burst pacing in control-FMT (*n* = 8) and ibrutinib-FMT rats (*n* = 10). (*H*) Number of rats in which atrial fibrillation (AF) could be reproducibly induced by right atrial burst pacing. (*I*) Atrial fibrillation duration in rats from control-FMT group (*n* = 8) and ibrutinib-FMT group (*n* = 10). (*J*) Atrial fibrillation duration in rats from control-FMT group (*n* = 8) and ibrutinib-FMT group (*n* = 10).

Our previous research demonstrated that ibrutinib can lead to an increased susceptibility to AF in rats.^35^ To explore the role of gut microbiota in ibrutinib-induced AF, we collected faecal samples from both control and ibrutinib-treated groups for 16S rRNA analysis. Principal coordinate analysis (PCoA) based on unweighted UniFrac distance revealed distinct microbiota clustering between the control and ibrutinib-treated rats (*Figure 1A*). The richness (as indicated by Chao1 indices) and the diversity (as indicated by Shannon and Simpson indices) of the gut microbiota exhibited no discernible disparity between the two groups (*Figure 1B–D*). At the phylum level, the relative abundance of *Bacteroidota* increased, whereas that of *Spirochaetota* decreased in rats undergoing ibrutinib treatment (*Figure 1E*). To investigate whether ibrutinib-induced gut microbiota dysbiosis confers pro-AF property, we conducted FMT experiments in rats. The group of rats received autogenous faecal microbiota is referred to the control-FMT group, while the group that was gavaged with faecal microbiota from ibrutinib-treated rats is designated as the ibrutinib-FMT group, as depicted in *Figure 1F*. Notably, we observed a substantial augmentation in AF inducibility among the rats in ibrutinib-FMT group (*Figure 1G* and *H*). Furthermore, the duration of AF was also significantly prolonged in the ibrutinib-FMT rats compared with control-FMT rats (*Figure 1I*), though the atrial effective refractory period did not display a noticeable difference between the two groups (*Figure 1J*). Taken together, our findings suggest that ibrutinib-induced AF is associated with gut microbiota dysbiosis.

### Oral supplementation of *Lactobacillus gasseri* protects rats from ibrutinib-related AF

**Figure 2:**
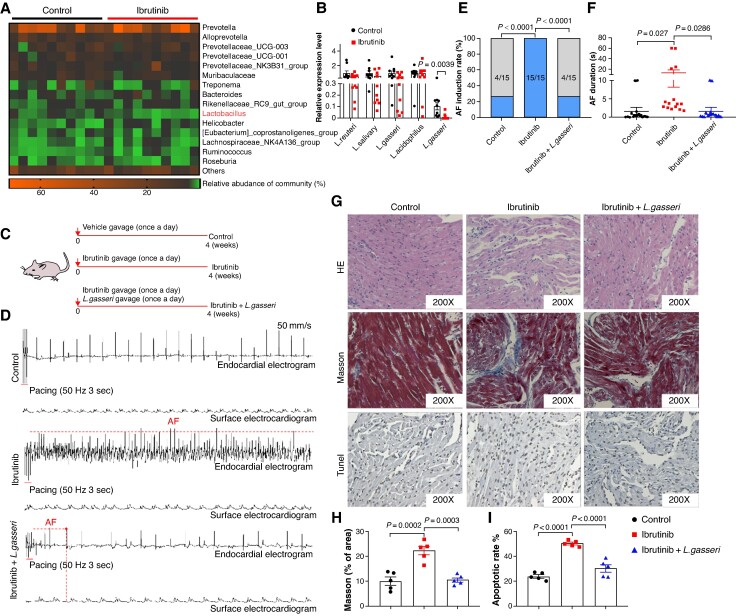
The supplementation of *L. gasseri* protects rats against ibrutinib-related atrial fibrillation (AF). (*A*) Heatmap showing the changes in the bacterial community of the control and ibrutinib group. (*B*) qPCR validation of the relative abundance of *Lactobacillus* in control and ibrutinib rats (*n* = 10). (*C*) Schematic illustration of ibrutinib and ibrutinib + *L. gasseri* gavage in rats. (*D*) Representative endocardial and surface electrograms recordings in response to burst pacing in control, ibrutinib, and ibrutinib + *L. gasseri* rats. (*E*) Number of rats in which AF could be reproducibly induced by right atrial burst pacing (*n* = 15). (*F*) Atrial fibrillation duration in rats from control, ibrutinib, and ibrutinib + *L. gasseri* group (*n* = 15). (*G*) Representative images of HE, Masson, and Tunel staining of rats (*n* = 6). (*H*) The collagen volume fraction in the atria of control, ibrutinib, and ibrutinib + *L. gasseri* rats (*n* = 6). (*I*) The ratio of Tunel-positive cells in control, ibrutinib, and ibrutinib + *L. gasseri* rats (*n* = 5).

The results of our 16S rRNA sequencing unveiled differential relative abundances of specific bacterial species between the control group and the ibrutinib group. Of particular note, we observed a significant reduction in the abundance of *Lactobacillus* in the ibrutinib group (*Figure 2A*). Confirming this, our PCR data indicated that among several common *Lactobacillus* species, only the abundance of *L. gasseri* showed a significant decrease in rats treated with ibrutinib (*Figure 2B*). *Lactobacillus gasseri*, a probiotic strain known to have health benefits to the host, has been identified as a potential target for the prevention and treatment of various diseases.^36^ In order to evaluate the potential of *L. gasseri* supplementation in preventing ibrutinib-related AF, we administered *L. gasseri* (1 × 10^9^ CFU, once a day for 4 weeks) to ibrutinib-treated rats (*Figure 2C*). Intriguingly, the ibrutinib-treated rats displayed a noteworthy augmentation in both AF inducibility and duration compared with the control rats. However, oral supplementation of *L. gasseri* prominently reduced both the AF inducibility (*Figure 2D* and *E*) and duration (*Figure 2D* and *F*) in the ibrutinib-treated rats. Concurrently, the ibrutinib-treated rats exhibited a disorganized arrangement of atrial myocytes, increased atrial fibrosis, and exacerbated apoptosis in atria tissues. In contrast, the rats receiving *L. gasseri* displayed well-aligned atrial myocytes, diminished fibrosis, and mitigated apoptosis (*Figure 2G–I*). In conclusion, supplementation with *L. gasseri* appears to alleviate atrial structural remodelling and prevent the onset of AF in rats treated with ibrutinib. In order to correlate the rat results with the clinical data of AF patients treated with ibrutinib, we compared changes in *L. gasseri* abundance in faecal samples from patients before taking ibrutinib with those who developed AF after taking ibrutinib. It was noted that the abundance of *L. gasseri* in faecal samples after 1 month of ibrutinib treatment decreased significantly compared with before ibrutinib treatment (see Supplementary material online, *Figure S1A*). These results suggest that the supplementation of *L. gasseri* may be clinical value in the treatment of ibrutinib-induced AF.

### *Lactobacillus gasseri-*derived butyrate alleviated ibrutinib-induced atrial structural remodelling

**Figure 3:**
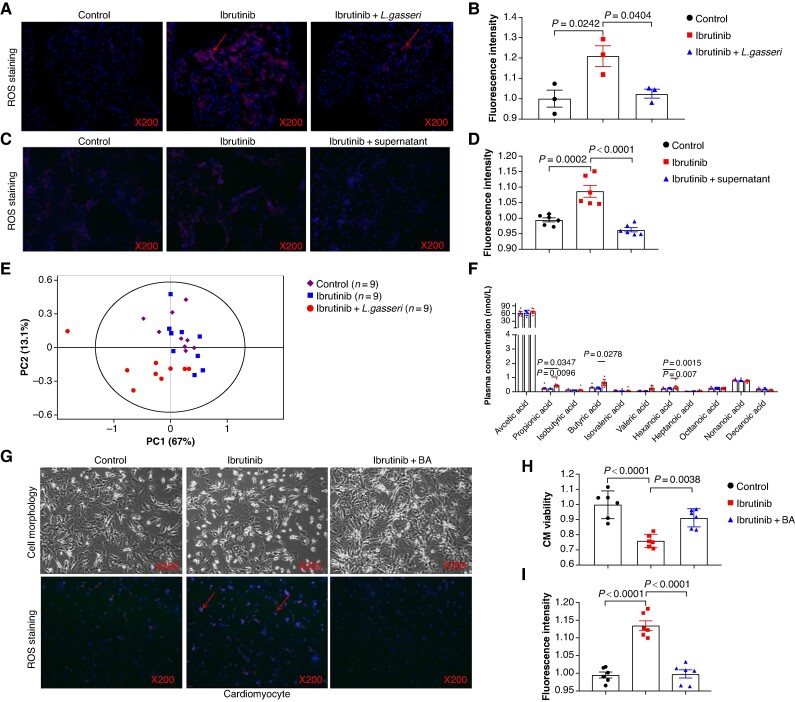
Butyric acid alleviated ibrutinib-induced elevation of reactive oxygen species (ROS) in cardiacmyocytes (CM). (*A*) Representative images of ROS staining in control, ibrutinib, and ibrutinib + *L. gasseri* atrial tissues. The generated ROS is marked by the arrow. (*B*) Fluorescence intensity of ROS staining of atrial tissues (*n* = 3). (*C*) Representative images of ROS staining in control, ibrutinib, and ibrutinib + supernatant atrial tissues (*n* = 3). (*D*) Representative images of ROS staining in control, ibrutinib, and ibrutinib + supernatant atrial tissues. (*E*) PCoA analysis of short-chain fatty acids in plasma from control, ibrutinib, and ibrutinib + *L. gasseri* rats (*n* = 9). (*F*) The plasma concentration of acetic acid, propionic acid, isobutyrate acid, butyrate (BA), isovaleric acid, valeric acid, hexanoic acid, heptanoic acid, octanoic acid, non-anoic acid, and decanoic acid in rats from control, ibrutinib, and ibrutinib + *L. gasseri* group. (*G*) Representative images of ROS staining in CM and representative optical microscope image showed the morphology of CM treated with control, ibrutinib, and ibrutinib + BA. The generated ROS is marked by the arrow. (*H*) The cell viability of CM (*n* = 6). (*I*) Cardiomyocyte fluorescence intensity of ROS staining (*n* = 6).

Oxidative stress plays a crucial role in the atrial structural remodelling and AF induced by ibrutinib.^37^ To explore whether the cardioprotective effect of *L. gasseri* operates through mitigating ibrutinib-induced oxidative stress, we measured the levels of reactive oxygen species (ROS) in the atrial tissues of rats. Rats in the ibrutinib group exhibited an increase in ROS levels within the atrial tissue. Conversely, the administration of *L. gasseri* attenuated the ibrutinib-induced rise in ROS (*Figure 3A* and *B*). Interestingly, ibrutinib did not trigger any significant increase in ROS levels within the ventricular tissue of the rats (see Supplementary material online, *Figure S2A* and *B*). Those findings indicated that *L. gasseri* can efficiently reduce the ROS escalation in atria induced by ibrutinib.

Gut microbiota have been reported to regulate the function of distant target organs via their metabolites.^38^ To evaluate whether *L. gasseri* exerts its beneficial effects through its metabolites, we exposed ibrutinib-pre-treated CM to the supernatant of *L. gasseri*. Intriguingly, the *L. gasseri* supernatant effectively attenuated the increase in ROS levels, and also counteracted the up-regulation of Bax and down-regulation of Bcl2 expression triggered by ibrutinib (*Figure 3C* and *D*; Supplementary material online, *Figure S3A* and *B*), indicating that *L. gasseri* may act through its metabolites. Metabolites derived from gut microbiota, including SCFAs, have been implicated in modulating mechanisms that promote AF.^5^ Short-chain fatty acids, as important metabolites for *L. gasseri*, were detected in the plasma of rats using targeted metabolomics. Interestingly, we observed elevated levels of propionic acid (PA), BA, and heptanoic acid in the plasma of rats administered with *L. gasseri*, compared with rats treated solely with ibrutinib (*Figure 3E* and *F*). Subsequently, we exposed primary rat CM and CFs to BA or PA the presence or absence of ibrutinib. Compared with the control group, the viability of CM in the ibrutinib group was significantly reduced. In contrast, BA treatment effectively enhanced CM viability (*Figure 3G* and *H*). Concurrently, CM treated with ibrutinib exhibited a decrease in the expression of the anti-apoptotic protein Bcl2 and an increase in the expression of the pro-apoptotic protein Bax. However, these changes were effectively reversed by BA treatment (see Supplementary material online, *Figure S4A* and *B*). Furthermore, we observed a notable increase in the number of ROS-positive cells in CM following stimulation with ibrutinib, which was reversed by BA treatment (*Figure 3G* and *I*). However, BA had no noticeable impact on the cell viability and ROS levels in ibrutinib-treated CF (see Supplementary material online, *Figure S5A* and *B*). There were no apparent alterations in ROS levels in the lung and liver tissues after treatment with ibrutinib or in conjunction with BA (see Supplementary material online, *Figure S6A*–*D*). At the same time, we also paid attention to the effect of ibrutinib on inflammation, we found that ibrutinib did not increase the expression of TNF-a, IL-6, and IL-1β in atrial tissue and in blood (see Supplementary material online, *Figure S7A*–*F*). Consistently, the levels of fibrosis-related proteins, TGF-β1 and α-SMA, remained unaltered following BA treatment (see Supplementary material online, *Figure S5C* and *D*). Building on our observations at the animal level, where supplementation with *L. gasseri* appeared to mitigate ibrutinib-induced atrial fibrosis in rats, we co-cultured CFs with supernatants from CM cultured with and without ibrutinib treatment. The viability of CFs exposed to supernatant from the ibrutinib-treated CM was significantly reduced compared with those exposed to control-CM supernatant (see Supplementary material online, *Figure S5E*). Furthermore, the levels of fibrosis-related proteins, TGF-β1 and α-SMA, were dramatically elevated (see Supplementary material online, *Figure S5F*). These findings suggest that ibrutinib indirectly influences CFs by altering CM metabolism. Taken together, *L. gasseri-*derived BA effectively counteracted ibrutinib-induced ROS accumulation and subsequent myocardial injury.

**Figure 4:**
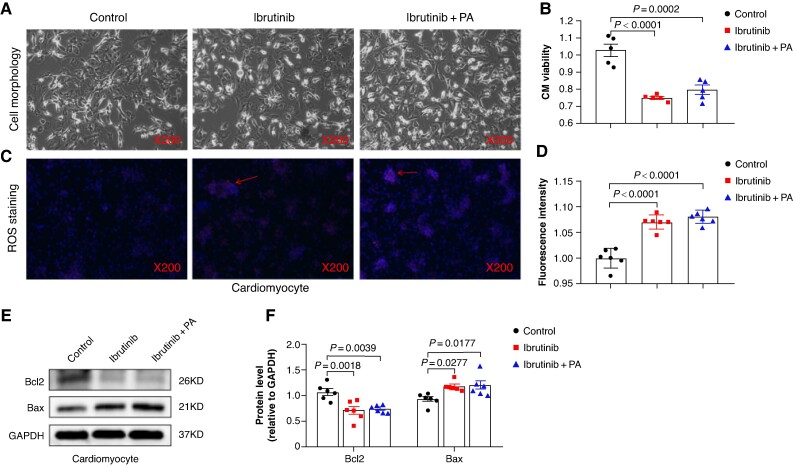
Propionic acid did not ameliorate ibrutinib-induced cardiomyocyte (CM) damage. (*A*) Representative optical microscope image showed the morphology of CM treated with control, ibrutinib, and ibrutinib + PA. (*B*) The cell viability of CM treated with control, ibrutinib, and ibrutinib + PA (*n* = 6). (*C*) Representative images of reactive oxygen species (ROS) staining of CM treated with control, ibrutinib, and ibrutinib + PA. The generated ROS is marked by the arrow. (*D*) The fluorescence intensity of ROS staining in CM (*n* = 6). (*E*) Representative bands of the protein levels of Bcl2 and Bax in CM treated with control, ibrutinib, and ibrutinib + PA (*n* = 6). (*F*) Quantification of the protein levels of Bcl2 and Bax in CM treated with control, ibrutinib, and ibrutinib + PA (*n* = 6).

To investigate whether PA exerts a similar cardioprotective effect, we evaluated the viability of CM in control, ibrutinib, and ibrutinib combined with PA groups. As shown in *Figure 4A* and *B*, no detectable improvement in CM viability was detected following PA supplementation. Moreover, we noted a significant increase in the number of ROS-positive cells in CM after ibrutinib exposure, which was not mitigated by PA supplementation (*Figure 4C* and *D*). Correspondingly, CM treated with ibrutinib exhibited a decrease in Bcl2 expression and an increase in Bax expression, while PA failed to reverse these changes (*Figure 4E* and *F*). Therefore, it appears that PA does not counteract ibrutinib-induced ROS accumulation and injury in CM.

### Butyrate prevents rats from ibrutinib-related AF

**Figure 5:**
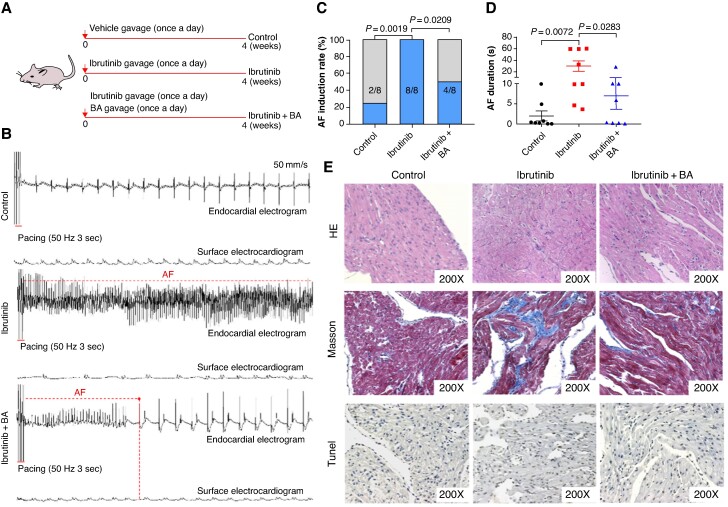
Supplementing butyric acid to improve atrial remodelling in rats treated with ibrutinib. (*A*) A schematic diagram for ibrutinib and ibrutinib + BA gavage in rats. (*B*) Endocardial and surface electrograms recordings in response to burst pacing in control, ibrutinib, and ibrutinib + BA group. (*C*) Number of rats in which atrial fibrillation (AF) could be reproducibly induced by right atrial burst pacing (*n* = 8). (*D*) Atrial fibrillation duration in rats from control, ibrutinib, and ibrutinib + BA group (*n* = 8). (*E*) Representative images of HE, Masson, and Tunel staining of rats (*n* = 5).

To evaluate the potential of BA in mitigating ibrutinib-related AF, we administered BA to rats by oral gavage once daily for 4 weeks. Rats gavaged with normal saline were designated as the control group, rats receiving ibrutinib and BA solvent were referred to the ibrutinib group, and rats receiving simultaneous gavage administration of both ibrutinib and BA were identified as the ibrutinib + BA group, as illustrated in *Figure 5A*. Notably, burst pacing commonly induced AF in the ibrutinib group, whereas it rarely triggered AF in the ibrutinib + BA group (*Figure 5B*). Both the inducibility of AF and its duration were noticeably reduced in the ibrutinib + BA group (*Figure 5C* and *D*). HE, Masson, and Tunel staining showed the disordered atrial tissue and elevated levels of fibrosis and apoptosis in rats treated with ibrutinib, while these detrimental effects were mitigated by the administration of BA (*Figure 5E*; Supplementary material online, *Figure S8A* and *B*). In conclusion, BA demonstrates potential in protecting rats from ibrutinib-induced AF by inhibiting the ibrutinib-induced atrial apoptosis and fibrosis.

### Ibrutinib inhibits the growth of *Lactobacillus gasseri* by increasing the production of ROS in Caco2 cells

**Figure 6:**
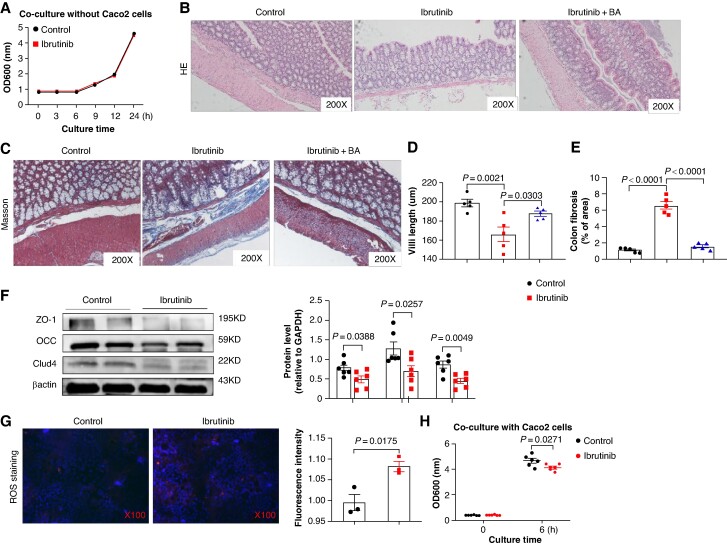
Ibrutinib accelerates intestinal damage, consequently inhibiting the growth of *L. gasseri*. (*A*) The graph depicts the OD600 changes within 24 h after the addition of *L. gasseri* to the control and ibrutinib group (*n* = 6). (*B*) Representative images of HE staining of proximal colon samples in control, ibrutinib, and ibrutinib + BA rats (*n* = 6). (*C*) Representative images of Masson staining of proximal colon samples in control, ibrutinib, and ibrutinib + BA rats (*n* = 6). (*D*) The villi lengths in control, ibrutinib, and ibrutinib + BA rats (*n* = 6). (*E*) The colon fibrosis area in control, ibrutinib, and ibrutinib + BA rats (*n* = 6). (*F*) Representative bands and quantification of the protein levels of ZO-1, OCC, and Clud-4 in colon samples of rats (*n* = 6). (*G*) The fluorescence of reactive oxygen species staining in Caco-2 cells treated with ibrutinib (*n* = 3). (*H*) The OD600 values of Caco-2 cells treated with ibrutinib for 6 h (*n* = 6).

To elucidate the mechanism by which ibrutinib induces a decrease in the abundance of *L. gasseri,* we monitored the growth of *L. gasseri* over a 24-h period in the absence or presence of ibrutinib by analysing OD600. Our results indicated that ibrutinib does not directly inhibit the growth of *L. gasseri* (*Figure 6A*). Given that the integrity of the intestinal barrier is a key determinant of gut microbiota colonization,^39^ we investigated if ibrutinib disrupts this integrity. We found that rats treated with ibrutinib had significantly shorter villi lengths in the proximal colon compared with control rats, as evidenced by HE staining (*Figure 6B* and *D*). Masson staining of proximal colon revealed a notable augmentation in the fibrotic area in ibrutinib rats; BA can ameliorate the level of colonic fibrosis induced by ibrutinib in rats (*Figure 6C* and *E*). Furthermore, the expression of ZO-1, claudin-4 (Clud-4), and occludin (OCC) was decreased in ibrutinib-treated rats relative to control rats (*Figure 6F*). We examined LPS levels in the intestine and blood, and noted that there were no alterations in LPS levels following treatment with ibrutinib (see Supplementary material online, *Figure S9A* and *B*). At the same time, FITC-dextran experiments were performed on rats to further test the permeability of the intestinal barrier after ibrutinib treatment. The concentration of FITC-dextran in serum was detected using a fluorescein microplate analyser, and we found that ibrutinib treatment did not increase the content of FITC-dextran in the serum of rats, indicating that ibrutinib would not cause serious damage to the intestinal barrier function (see Supplementary material online, *Figure S9C*). These results suggest that while the expression of tight junction proteins such as ZO-1, OCC, and Clud-4 in the intestines is down-regulated following ibrutinib treatment, ibrutinib does not cause serious damage to the intestinal barrier function. The Caco-2 cell line, derived from human colorectal adenocarcinoma, is extensively utilized in diverse mechanistic studies. We then measured ROS levels in Caco-2 cells following ibrutinib treatment, and found an increase in ROS levels (*Figure 6G*). Subsequently, we performed OD600 detection on Caco-2 cells co-cultured with *L. gasseri* for 6 h following 48 h of ibrutinib treatment. We observed a reduction in *L. gasseri* growth after ibrutinib administration, suggesting that escalated production of ROS induced by ibrutinib can impede the proliferation of *L. gasseri* (*Figure 6H*).

## Discussion

This study provides novel pathophysiological insights into the aetiological association between gut microbiota dysbiosis and ibrutinib-related AF. Herein, we revealed that ibrutinib disturbed gut microbiota, which confers pro-AF property to rats. Notably, the abundance of *L. gasseri* was significantly decreased in rats treated with ibrutinib, and oral supplemention with *L. gasseri* protected rats from ibrutinib-related AF. Mechanistically, *L. gasseri*-derived BA mitigates atrial structural remodelling by inhibiting the generation of ibrutinib-induced ROS, thereby demonstrating its protective effects against ibrutinib-associated AF. Taken together, we discovered a previously unrecognized role of *L. gasseri* in impeding the progression of ibrutinib-associated AF, suggesting that *L. gasseri* may be a potential target for the prevention and treatment of ibrutinib-induced AF.

Atrial fibrillation is one of the most common cardiac arrhythmia encountered in clinical practice, and is associated with substantial morbidity and mortality.^40^ Pathophysiological mechanisms underlying AF include both structural remodelling and electrophysiological changes.^41^ Previous research attributed ibrutinib-mediated AF to inhibition of C-terminal Src kinase.^42^ Ibrutinib has been demonstrated to promote AF by disrupting mitochondrial quality control mediated by A-kinase anchoring protein 1 (AKAP1)-mediated mitochondrial quality surveillance in CM, and the AKAP1 activation can be employed to prevent and treat ibrutinib-induced AF.^43^ With increasing focus on gut microbes and understanding of the gut–heart axis, more and more studies suggest that changes in gut microbes may provide a new direction for the treatment of cardiovascular diseases such as AF. Our research delves into the mechanisms of ibrutinib-related AF from the perspective of gut microbes, offering a supplementary understanding of AF aetiology. The intricate relationship between gut microbiota and drug efficacy and toxicity has become a burgeoning field of research. The gut microbiota, a complex community of microorganisms inhabiting the human gastrointestinal tract, has been reported to modulate the efficacy and toxicity of multiple anti-tumour drugs. Akkermannia, *Bifidobacterium adolescentis, Bifidobacterium longum,* and *B. fragilis* can enhance the efficacy of immune checkpoint inhibitors. *Prevotellaceae* alleviates PD-1/PD-L1 inhibitor-related cardiotoxicity through BA.^20^ In the present study, we revealed that ibrutinib caused an increase in the susceptibility to AF in rats, and this pro-AF property can be transferred through ibrutinib-related FMT. The link between gut microbiota dysbiosis and AF has been established in both rodent models and human subjects.^5^ The dysbiotic signature of AF is characterized by an imbalance in gut microbiota, particularly a depletion of SCFA-producing bacteria.^24^ In this study, we verified that rats subjected to ibrutinib treatment exhibited a dysbiotic state characterized by a notable decline in the abundance of BA-producing bacteria, specifically *L. gasseri.* Those findings suggested that ibrutinib-induced microbiota dysbiosis is responsible for heightened AF risk.

*Lactobacillus gasseri,* as a beneficial BA-producing bacterium, elicits diverse health benefits through its anti-microbial activity, bacteriocin synthesis, and immunomodulatory effects on the innate and adaptive systems.^44^ It was reported that *L. gasseri* JM1 mitigated intestinal barrier impairment in colitis mice by modulating the inflammatory cytokines, gut microbiota, and SCFAs.^45^  *Lactobacillus gasseri* RW2014 ameliorates hyperlipidemia by orchestration of the bile acid metabolism and gut microbiota composition in rats.^46^ In our study, we found that supplementation of *L. gasseri* substantially reduced the AF induction rate and AF duration in rats treated with ibrutinib, suggesting that *L. gasseri* potentially assumes an important role in the pathological progression of ibrutinib-associated AF.

Gut microorganisms regulate host metabolism is mainly through the synthesis of SCFAs and other metabolites.^47^ When the gut microbiota experiences dysfunction, the delicate balance between bacteria and the host is disturbed, resulting in the accumulation of endotoxin and the reduction in SCFAs.^48^ The majority of SCFAs exert beneficial effects in the regulation of related diseases. Butyrate, as SCFAs, exerts various beneficial effects on the human body. It is primarily produced by microbes through the fermentation of dietary fibres. Supplementation with BA salts can be achieved by consuming fibre-rich foods such as vegetables, fruits, and grains. As reported, consumption of *L. gasseri* CECT5714 and *L. coryniformis* CECT5711 daily for 4 weeks was associated with a notable augmentation in BA level in the human intestine.^49^ Butyrate and PA have been demonstrated to lower blood pressure,^50^ mitigate ischaemia/reperfusion injury,^50,51^ and decrease the risk of coronary artery disease^52,53^ and atherosclerosis.^54^ Interestingly, we found that BA supplementation can significantly reduce the ROS level of atrial myocytes, enhance the viability of CM, and ameliorate apoptosis in rats subjected to ibrutinib treatment. In addition, studies indicate that oral and dietary supplementation of BA salts has been shown to prevent obesity and insulin resistance induced by high-fat diet.^55–57^ Moreover, Gibson and Rosella isolated colonic crypt cells from patients diagnosed with colorectal cancer, Crohn’s disease, or ulcerative colitis and assessed IL-8 secretion in response to BA treatment (1 mmol/L) over a 24 h time course; in all disease groups BA administration significantly lowered IL-8 concentrations in comparison to control,^58^ demonstrating that BA has potent anti-inflammatory properties and an inhibitory effects on colorectal cancer.^57,59^ Furthermore, there are already BA supplements available on the market that can be used to restore gut function, alleviate constipation, and improve colitis.

## Limitations

There are several limitations in our study. During the course of the experiment, due to experimental design constraints, we did not conduct the gut microbiota analysis after FMT to confirm re-colonization. Furthermore, the AF inducibility between ibrutinib treated rats and control-FMT, as well as between ibrutinib treated rats and ibrutinib-FMT, warranting further explorations. In terms of clinical translation, first, although we observed a decrease in the abundance of *L. gasseri* in the faeces of patients undergoing ibrutinib treatment with AF, the administration of *L. gasseri* has not been validated in human subjects, which yields therapeutic value of *L. gasseri* is currently based on animal studies. Secondly, when considering the application of new probiotic species, it is crucial to establish proper identification and gather comprehensive *in vitro*, *in vivo,* and clinical evidence to support their utilization in food or supplements for safe consumption by the greater population.^60^ There is an increasing body of evidence that indicates *L. gasseri* has significant potential for probiotic application by fulfilling the screening criteria for probiotic microorganisms^44^ and *L. gasseri* indicates the absence of any association with a health detriment affirms its safety.^61^ Whether *L. gasseri* can be used for the prevention of ibrutinib-associated AF in patients needs to be further explored. Therefore, prospective multi-centre and large sample clinical researches are urgently needed to further validate those findings.

## Conclusions

In the present study, we elucidate a mechanistic link between gut microbiota dysbiosis and ibrutinib-related AF. Transplantation of *L. gasseri* and supplementation with its metabolite, BA, notably attenuated atrial structural remodelling and susceptibility to AF. These results underscore the potential of *L. gasseri* and its beneficial metabolites as a therapeutic approach to alleviate ibrutinib-induced AF.

## Ethics approval and consent to participate

All animal protocols were performed in accordance with the Guide for the Care and Use of Laboratory Animals and were approved by the Animal Care and Utilization Committee of Harbin Medical University.

## Supplementary Material

euaf018_Supplementary_Data

## Data Availability

The data underlying this article will be shared on reasonable request to the corresponding author.
