# Supplementary material for: Lactobacillus gasseri prevents ibrutinib-associated atrial fibrillation through butyrate
Source: Europace. 2025 Jan 17;27(2):euaf018. doi: 10.1093/europace/euaf018 (PMC11795659; doi:10.1093/europace/euaf018)
Supplement: euaf018_Supplementary_Data [file euaf018_supplementary_data.docx]

**Supplement Figure**

**
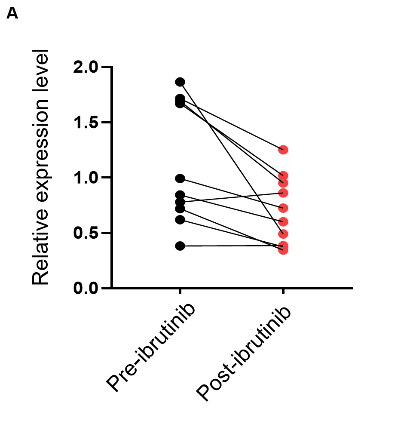
**

**Supplement Figure1. Patients treated with ibrutinib had reduced *L.gasseri* abundance in the fecal. (A)** The variation in the abundance of *L.gasseri* in the fecal samples of the same patient before and after ibrutinib treatment (n=10).

**
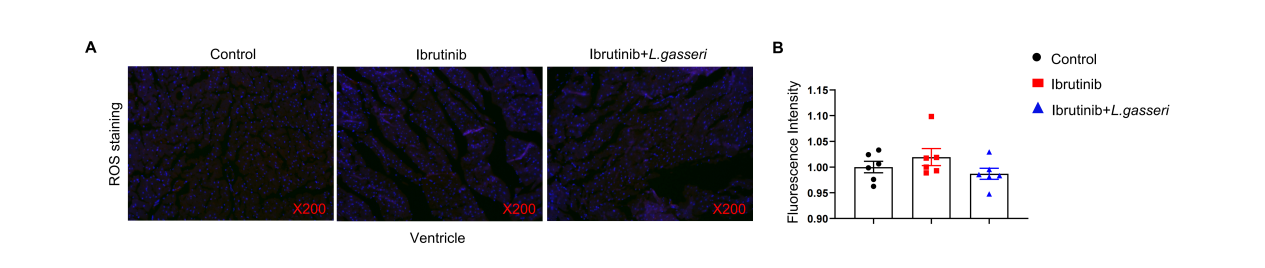
**

**Supplement Figure2. Ibrutinib does not increase the production of ROS in ventricular tissue. (A)** Representative images of ROS staining in ventricular tissue of control, ibrutinib and ibrutinib+ *L.gasseri* rat (n=6). **(B)** Fluorescence intensity of ROS staining of control, ibrutinib and ibrutinib+ *L.gasseri* rat (n=6).


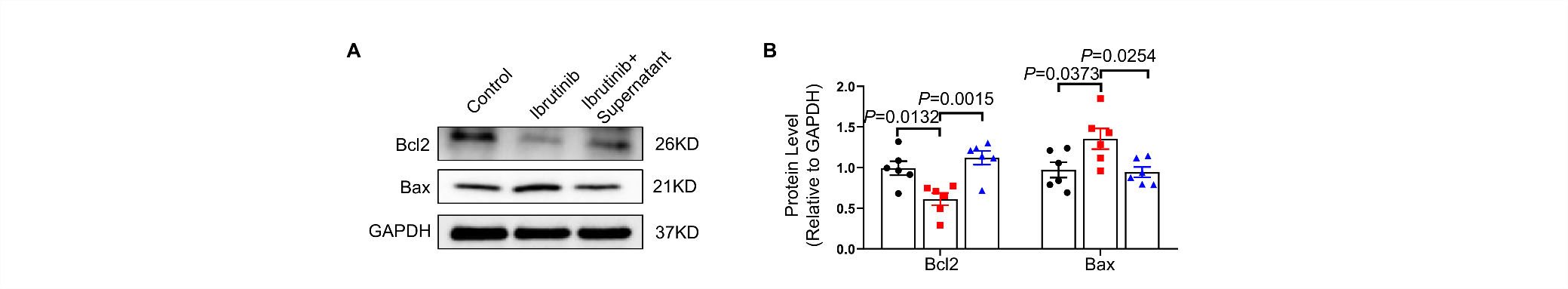


**Supplement Figure3.** **The supernatant of *L.gasseri* alleviates ibrutinib-induced apoptosis of cardiomyocytes.**  **(A-B)** Representative bands and quantification of the protein levels of Bcl2 and Bax in cardiomyocytes (n = 6).


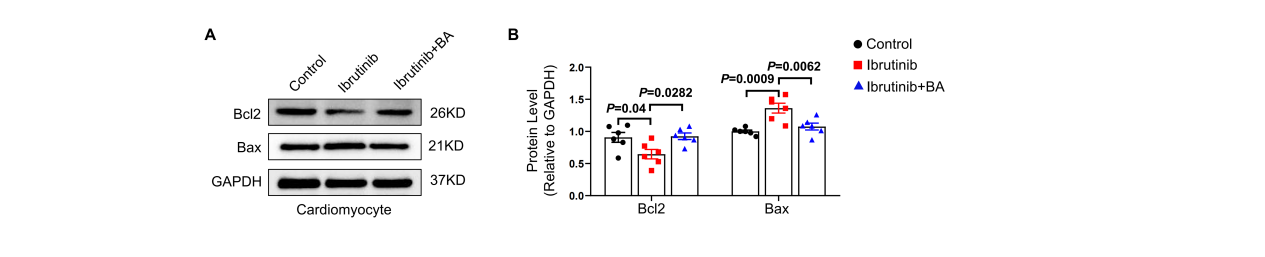


**Supplement Figure4.** **BA alleviates ibrutinib-induced apoptosis of cardiomyocytes.**  **(A-B)** Representative bands and quantification of the protein levels of Bcl2 and Bax in cardiomyocytes (n = 6).


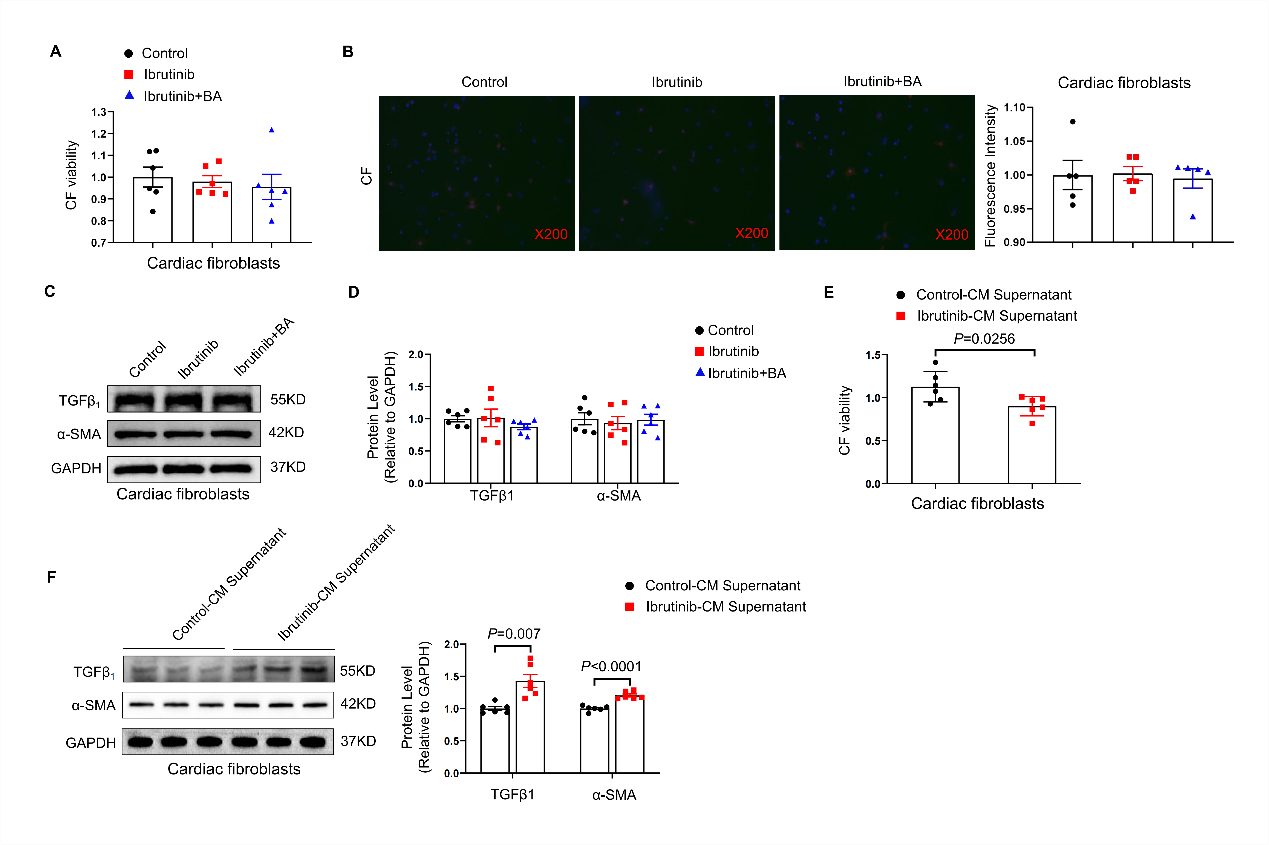


**Supplement Figure5.** **BA does not alter the viability of cardiac fibroblasts or the generation of ROS induced by ibrutinib.** **(A)** The cell viability of CF treated with control, ibrutinib and ibrutinib+BA (n = 6). **(B)** Representative images of ROS staining of CF treated with control, ibrutinib and ibrutinib+BA (n=6). **(C-D)** Representative bands and quantification of the protein levels of TGF-β1 and α-SMA in CF (n = 6). **(E)** The cell viability of CF treated with control-CM supernatant and ibrutinib-CM supernatant (n = 6). **(F)** Representative bands and quantification of the protein levels of TGF-β1 and α-SMA in CF (n = 6).


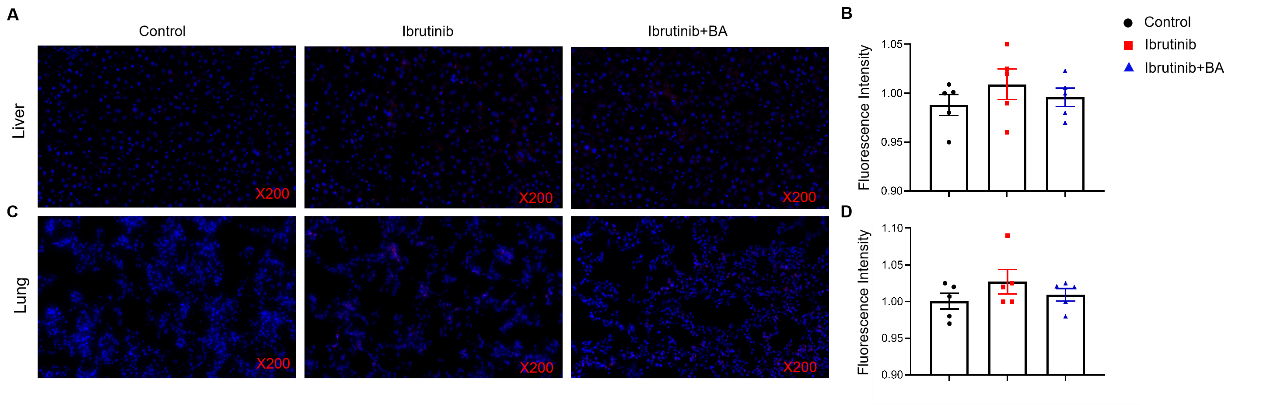


**Supplement Figure6.** **Ibrutinib did not increase ROS in the lung and liver of rats. (A)** Representative images of ROS staining in control, ibrutinib and ibrutinib+BA liver tissues. **(B)** Fluorescence intensity of ROS staining of liver tissues (n=6). **(C)** Representative images of ROS staining in control, ibrutinib and ibrutinib+BA lung tissues. **(D)** Representative images of ROS staining in control, ibrutinib and ibrutinib+BA lung tissues (n=6).

**
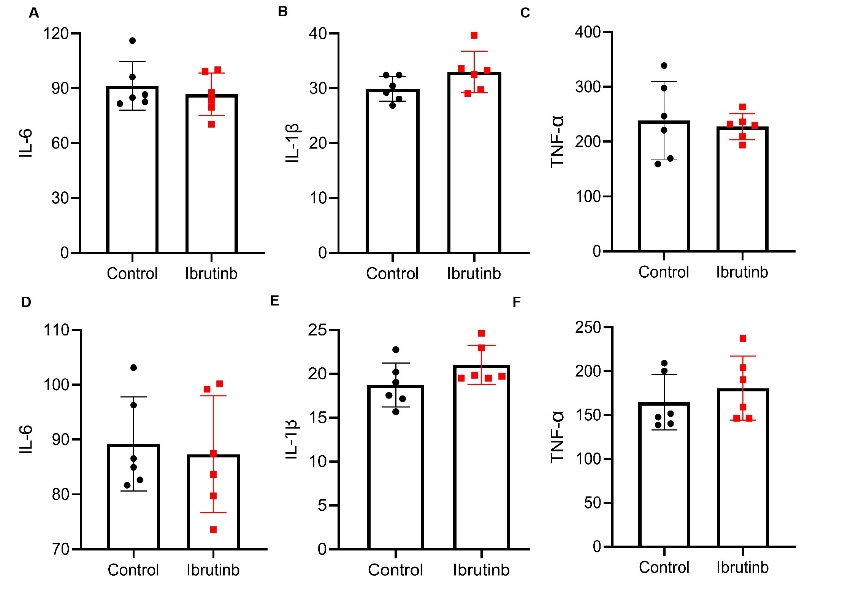
**

**Supplement Figure7. Ibrutinib** **did not increase the expression of TNF-a, IL-6 and IL-1β in atrial tissue. (A)** - **(C)** Expression of TNF-a, IL-6 and IL-1β in atrial tissue of control and ibrutinib groups (n=6). **(D)** - **(F)** Expression of TNF-a, IL-6 and IL-1β in the blood of control and ibrutinib groups (n=6).


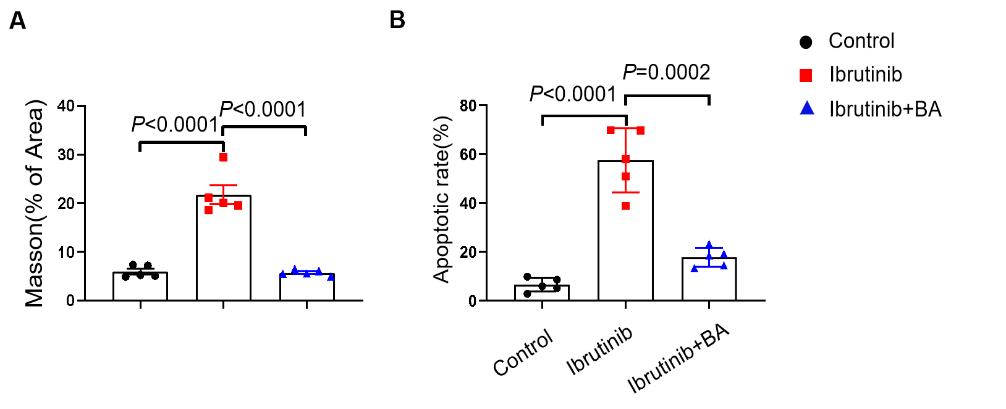


**Supplement Figure8. BA ameliorates the extent of atrial fibrosis and apoptosis induced by ibrutinib. (A)** The atrial fibrosis (blue) in control, ibrutinib and ibrutinib+BA rats (n = 5). **(B)** The ratio of Tunel-positive cells in control, ibrutinib and ibrutinib+BA group (n =5).


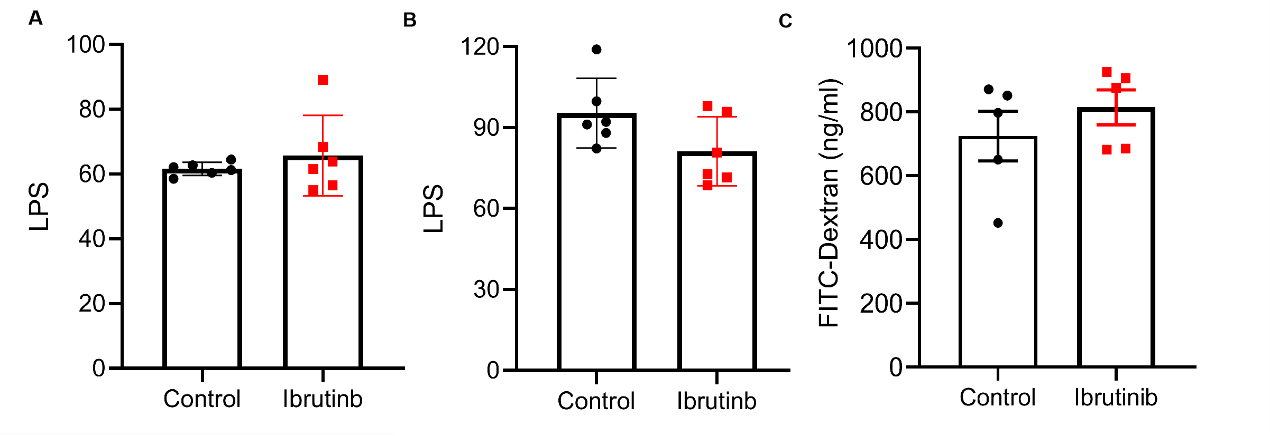


**Supplement Figure9. Ibrutinib did not induce severe damage to the intestinal barrier. (A)** The levels of LPS in the blood of rats in the control and ibrutinib group (n =6). **(B)** The levels of LPS in the intestines of rats in the control group and ibrutinib group (n =6). **(C)** Content of FITC-dextran in serum in the control and ibrutinib group (n = 6).

**TableS1**

|  | Forward primer (5’-3’) | Reverse primer (3’-5’) |
| --- | --- | --- |
| 16s | TGATGCACTTGCAGAAAACA | ACCAGAGGAAATTTTCAATAGGC |
| *L. gasseri* | AAGGGCGCACGGTGAATGCCT | TGCTATCGCTTCAAGTGCTT |
| *L. reuteri* | GCCGCCTAAGGTGGGACAGAT | AACACTCAAGGATTGTCTGA |
| *L. salivary* | ATATCTCTAAGGATCGCATG | CCGTGTCTCAGTCCCAATGT |
| *L. johnsonii* | GACCAGCGCACCAAGTGATA | AGCGTAGCGTTCGTGGTAAT |
| *L.acidophilus* | CACCGCTACACATGGAG | AGCAGTAGGGAATCTTCCA |
